# It’s a Matter of Perspective: The Role of Aging Expectations and Self-Efficacy Towards Engagement in Healthy Lifestyles Among Older Adults

**DOI:** 10.31372/20190403.1057

**Published:** 2019

**Authors:** Johnny Julvesano Yao

**Affiliations:** Velez College, Cebu, Philippines

**Keywords:** aging expectations, subjective aging, healthy behaviors, health promotion, healthy aging, self-efficacy

## Abstract

This study aimed to investigate whether aging expectations predict engagement in healthy lifestyles. Furthermore, it aimed to determine whether self-efficacy mediates the relationship between aging expectations and engagement in healthy lifestyles. This study enlisted 95 respondents who were 60 years old and older in a large metropolitan area in the Philippines. A four-part instrument package was utilized to measure the respondent’s (1) demographic profile, (2) aging expectations using the Expectations Regarding Aging (ERA-12) Survey, (3) engagement in healthy lifestyles using Health-Promoting Lifestyle Profile II (HPLP-II), and (4) self-efficacy using the Self-efficacy for Self-direction in Health Scale. Data were analyzed using linear regression and mediation analysis. Results show that aging expectations and self-efficacy predicted engagement in healthy lifestyles. Moreover, self-efficacy was found to be a significant mediator between the variables. Programs that promote a positive aging expectation and high self-efficacy should be pursued by the government and non-government organizations.

## Introduction

As the population of older individuals steadily increases, there is also an increasing body of literature focused on exploring factors related to successful aging. It is vital for the health care community to support older adults attain optimum aging and understand how older adults expect and sustain physical and mental function. If older adults feel that health problems are an expected part of aging, they might be less willing to join in health-promoting and disease preventive behaviors that make successful aging possible. For instance, health problems that are attributed to old age have been related to lower rates of preventive care and delays in treatment seeking (Sarkisian, Hays, Berry, & Mangione 2002). Older adults who have negative aging expectations seek less healthcare for manageable problems. Therefore, changing older adults’ aging expectations may significantly increase the proportion of older adults getting care for these health problems that have a negative consequence on the quality of life of older persons. However, for some older adults with health problems that cannot be cured, having low aging expectations may express a realistic coping mechanism (Calman 1984; Clark 1995; Keller et al., 1989; as cited in Sarkisian et al., 2002), and intervening to change expectations could be pointless.

Modifiable health behaviors have a significant role in the improvement and maintenance of health and well-being. One health behavior that has been extensively researched is physical activity. Numerous studies have suggested that physical activity is vital to the health of older adults. Earlier research suggests that people who keep negative aging expectations underestimate their capacity to participate in physical activity, consequently accepting a more inactive lifestyle (O’Brien Cousins, 2000, 2003). Similarly, older persons who believe that old age would result in unavoidable physical decline did not engage in physical activity (O’Brien Cousins, 2000, 2003). Sarkisian, Steers, Hays, and Mangione (2005) studied the association between aging expectations and physical activity and found a significant positive relationship between positive aging expectations and aerobic activity. People with negative aging expectations were less likely to engage in physical activity. Therefore, negative aging expectations may be a hindrance to physical activity in older adults (Sarkisian et al., 2005).

Self-perception of aging can also affect a person’s engagement in other health-promoting behaviors such as participating in exercise, eating a healthy balanced diet, use of health care resources, having regular physical examinations, and limiting the use of alcohol and/or tobacco (Levy & Myers, 2004). Moreover, people’s views about their capacity to succeed have a strong impact on their actions. According to Bandura (as cited in Brown, 2015), “people with high self-efficacy beliefs think they have the ability to succeed at a task, to overcome obstacles, and to reach their goals.”

Resnick, Palmer, Jenkins, and Spellbring (2000) studied self-efficacy of older adults towards their ability to engage in physical activity regardless of barriers and challenges. Their research established the importance of older adults’ self-efficacy towards engagement in physical activity in addition to the knowledge of the health benefits exercise can deliver. Research by Barriopedro and colleagues (as cited in Singh, Shukla, & Singh 2010) found that older adults who have high self-efficacy and participated in physical activity reported lower levels of depression and increased levels of life satisfaction (Barriopedro et al., as cited in Singh et al., 2010). Furthermore, people will select a behavior grounded on the expectation about the capability to do a given action and this perceived capability plays a significant role in improving mental well-being & functioning and exercise adherence (Davis et al., as cited in Singh et al., 2010).

There is limited evidence that investigates the mediating effect of self-efficacy between the aging expectations and engagement in healthy lifestyles. It is hypothesized that aging expectations would influence self-efficacy and subsequently influence health behaviors among older adults. Identifying the relationship between expectations regarding aging and health behaviors is therefore of vital importance to clients, nurses, other health care professionals, and policymakers concerned in creating goals of care that will enhance successful aging. This study aimed to determine whether aging expectations predict engagement in healthy lifestyles. Furthermore, the study sought to determine whether self-efficacy mediates the relationship between aging expectations and engagement in healthy lifestyles.

## Methodology

The study was approved by the Velez College ethics review committee prior to gathering the data. Respondents were individuals who are 60 years old and above. There were a total of ninety-five respondents aged 60 to 85 years old. The computed sample size was ninety based on a power analysis using G-power software (power = 0.90; *α* = 0.05; medium effect size = 0.15). Respondents were from a large metropolitan area in the Philippines through convenience sampling. Participants provided written informed consent prior to initiating the study. Distribution of the questionnaire was done during the monthly meetings of the local senior citizen’s association. Since not all may have joined the monthly meetings, the questionnaire was also distributed in their respective homes with the assistance of community personnel to enhance representativeness. This was either through face-to-face interviews or through direct individual or group administration, depending on their preference or their capabilities.

The main research instruments used for this study were three (3) standardized tools, namely: Expectations Regarding Aging (ERA-12) Survey, Health-Promoting Lifestyle Profile II (HPLP-II), and Self-efficacy for Self-direction in Health Scale. All the tools were translated to local dialect and were validated by two nursing clinical instructors whose expertise are in community health nursing and gerontology nursing for semantic equivalence.

The first part of the questionnaire obtained the demographic profile of the respondents including age, sex, marital status, educational attainment, and employment status. The second part of the questionnaire was the Expectations Regarding Aging (ERA-12). It is a twelve-item survey that measures expectations regarding aging with three 4-item scales (expectations regarding physical health, expectations regarding mental health, and expectations regarding cognitive function), and one global expectations regarding aging scale combining all twelve items. The ERA-12 scales demonstrated acceptable levels of reliability and construct validity in two very different samples of community-residing older adults (*n* = 429; *α* = 0.88 and *n* = 643; *α* = 0.89). Though substantially shorter, the ERA-12 captures 88% of the variation in the ERA-38 overall score (Sarkisian et al., 2005). The ERA-12 was chosen over the full thirty-eight item ERA survey since it may not be practical to use the longer, original version. The statements are followed by four responses: “Definitely True,” “Somewhat True,” “Somewhat False,” and “Definitely False.” Possible scores range from 0 to 100, with higher scores indicative of expecting achievement and maintenance of high physical and mental functioning with aging (for self and others), and low scores indicate expecting decline with aging. In addition, there are no cut points for what is optimal.

The third part of the questionnaire was the Health-Promoting Lifestyle Profile II (HPLP-II) which consists of 50 questions related to current engagement in health-promoting lifestyle factors (Walker, Sechrist, & Pender, 1995). These lifestyle factors are quantified using six subscales (i.e., Health Responsibility, Physical Activity, Nutrition, Spiritual Growth, Interpersonal Relations, and Stress Management). The construct validity of these subscales was analyzed using factor analysis, which confirmed the six-dimensional structure of the HPLP-II (Walker et al., 1995). The HPLP-II has an alpha coefficient of .94, and the subscales have alpha coefficients ranging from .79 to .87, suggesting that the measure and its subscales are internally consistent (Walker et al., 1995). A total score can also be calculated by scoring responses to all of the items on the survey (i.e., items from every subscale). Each statement on the survey is followed by four responses: “Never,” “Sometimes,” “Often,” and “Routinely”. Individual responses are scored on a one to four scale, with the overall score being obtained by averaging all of the responses. For the purpose of this study only four subscales were used, namely: Health Responsibility, Physical Activity, Nutrition, and Stress Management, which reduced the number of items to thirty-four.

The fourth part of the questionnaire was the Self-efficacy for Self-direction in Health Scale. The tool evaluates the extent older adults have confidence in taking good care of their health. It has four dimensions: physical exercise, healthy diet, engaging in health-related learning, and visits to health professionals (Oliveira, Silva, & Lima 2016). It features on the first page an exercise of familiarization with the response scale, which varies between 0 (cannot do at all) and 10 (totally certain can do), containing the numbers in large font size. The response scale, and its sixteen items, begin with the statement “I am confident that I am able, by myself…”. The Cronbach’s alpha (*α* = 0.87) obtained is, overall, quite suitable for global reliability. The tool has enough psychometric strength and adequacy for applied use in the field of health of older adults, more specifically when assessing to what extent these people have confidence in their ability to take care of their health (Oliveira et al., 2016).

The data collected was analyzed using IBM SPSS statistical software version 23. Descriptive statistics including means and standard deviations were calculated for the continuous variables and percentages and frequencies for categorical variables. Linear regression and mediation analyses using PROCESS version 3 (Hayes, 2017) were applied to analyze the data for the main variables.

## Results

Table 1 shows that majority of the participants were categorized as: young old (60–69 years), females, widow/widower, unemployed, and college level or college graduates aged 60 to 85 years old. The mean age of the participants was 68 years old (SD = 6.6).

**Table 1:** Profile of Participants (n = 95)

Profile	*f*	%
Age		
Young old (60–69)	59	62
Middle old (70–79)	30	32
Oldest old (≥80)	6	6
Gender		
Male	18	19
Female	76	81
Marital status		
Single	5	5
Married	41	43
Widow/widower	43	46
Separated	6	6
Employment status		
Employed	10	10
Unemployed	85	90
Educational attainment		
No formal education	1	1
Elementary level	10	10
Elementary graduate	11	12
High school level	12	13
High school graduate	15	16
College level	21	22
College graduate	21	22
Post graduate	4	4

To test if expectations regarding aging and self-efficacy in health significantly predict-health-promoting lifestyle, a regression analysis was done. Table 2 shows the prediction model that shows path b and c′ is statistically significant, *F*(2, 92) = 11.16, *p* < .001 and shows that the regression model has an *R*^2^ of .25. This means that about 25% of the variability of the dependent variable, which is health-promoting lifestyle, is predicted by the independent variables included in the study. The remaining 75% of the variability in the dependent variable is still unaccounted for and may be caused by other variables or external factors that were not included in the study.

Expectations regarding aging and self-efficacy in health were used in the regression analysis to predict health-promoting lifestyles. The unstandardized and standardized regression coefficients of the predictors are also shown in Table 2.

Table 2 indicates that the independent variables—expectations regarding aging (*β* = .28, *p* = .012) and self-efficacy in health (*β* = .35, *p* = .002) are significant predictors to the dependent variable, health-promoting lifestyle. Both expectations regarding aging and self-efficacy in health have positive coefficients, which means that an increase or decrease of its value will subsequently increase or decrease the level of health-promoting lifestyles, respectively. Further, it means that for every one-point increase in the independent variables there will be a corresponding increase equivalent to the value of its beta coefficient in the level of health-promoting lifestyle. Finally, self-efficacy in health received the strongest weight in the model followed by expectations regarding aging.

**Table 2:** Regression and Mediation Analysis of Expectation Regarding Aging, Self-Efficacy in Health, and Health-Promoting Lifestyle

Path model	*R*^2^	*F*	*B*	*SE (B)*	95% CI	*β*
Path b and c′: DV = health-promoting lifestyle	.25	11.16∗∗∗				
Path b: IV = self-efficacy in health			.10∗∗	.03	[0.04, 0.16]	.35∗∗
Path c′: IV = expectations regarding aging			.26∗	.10	[0.06, 0.47]	.28∗
Path c: DV = health-promoting lifestyle	.14	10.80∗∗				
IV = expectations regarding aging			.34∗∗	.11	[0.14, 0.55]	.37∗∗
Path a: DV = self-efficacy in health	.06	4.61∗				
IV = expectations regarding aging			.83∗	.38	[0.06, 1.60]	.25∗
Indirect effect: DV = health-promoting lifestyle						
IV = expectations regarding aging			.08†	.05	[0.01, 0.20]	.09†
Mediator = self-efficacy in health						

*Note*. *n* = 95. *B*: unstandardized beta; *SE*: standard error; CI: confidence interval; *b*: standardized beta; DV: dependent variable; IV: independent variable.

∗ *p* < .05.

∗∗ *p* < .01.

∗∗∗ *p* < .001.

† Significant indirect effect.

**Figure 1:**
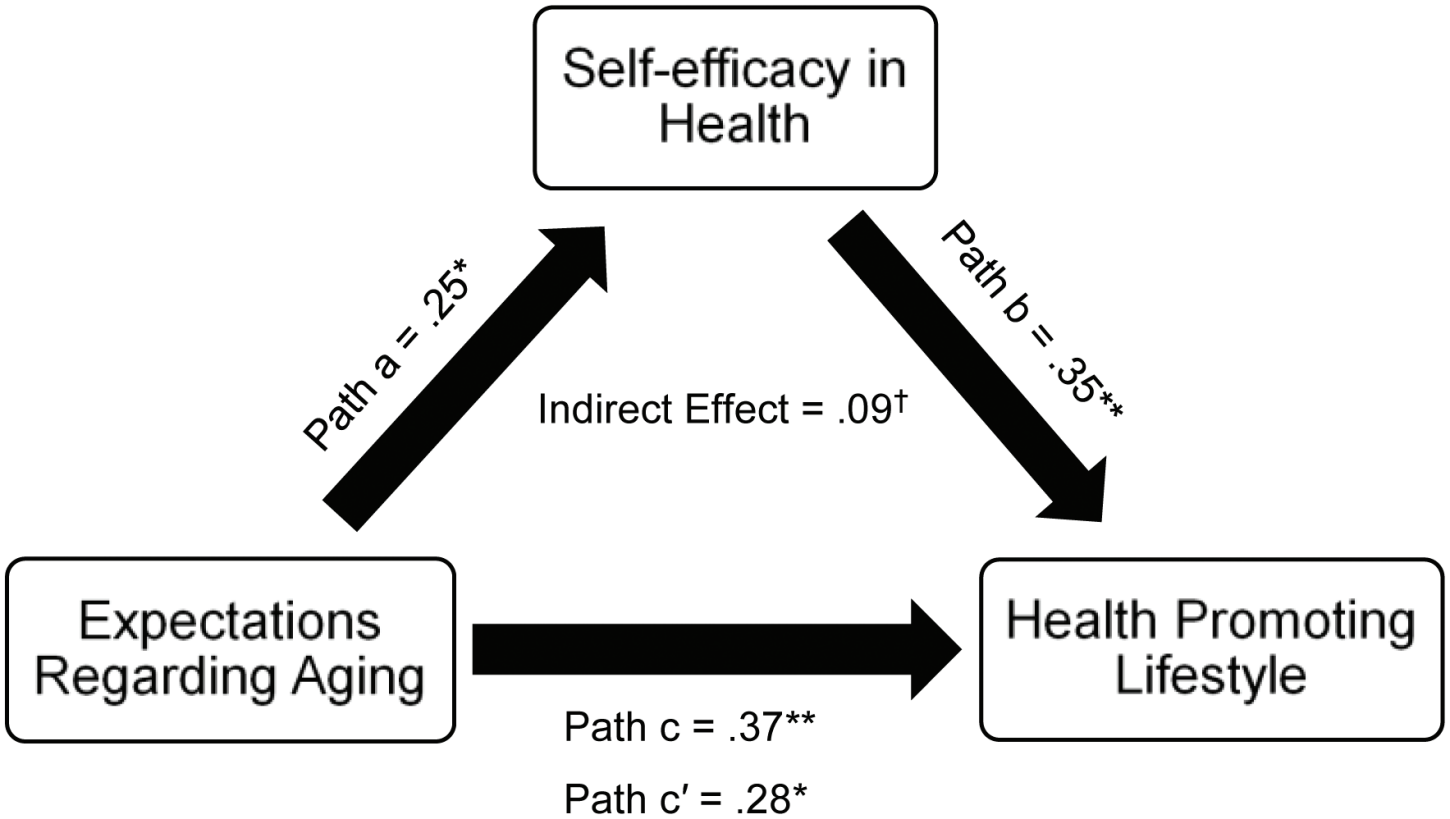
Mediation model of expectations regarding aging, self-efficacy in health, and health-promoting lifestyle. Standardized beta coefficients are shown. ∗*p* < .05. ∗∗*p* < .01. †significant indirect effect.

To determine if self-efficacy mediated the relationship between expectations regarding aging and health-promoting lifestyle, a mediation analysis was done. The mediation analysis is summarized in Table 2 and illustrated in Figure 1 to show the paths of the relationships between the variables.

The total effect of the independent variable, expectations regarding aging, to the dependent variable, health-promoting lifestyle, is shown as path c. This is the effect without the mediator included. As shown in Table 2, expectations regarding aging’s total effect is significant (*β* = .37, *p* = .002). In comparison, path c′ shows the direct effect of the independent towards dependent variable with the inclusion of the mediator variable. Table 2 and Figure 1 shows that it is significant (*β* = .28, *p* = .012), but the direct effect is less than the total effect (c′: *β* = .28 < c: *β* = .37). This reduction in the beta coefficient means that including path b as a mediator may have influenced the effects of the independent variable.

Furthermore, path a illustrates that the independent variable, expectations regarding aging, is a significant predictor of the mediator variable, self-efficacy in health (*β* = .25, *p* = .035). This may also suggest that mediation is present in the model. This would suggest that the independent variable could affect the dependent variable through its effect on the mediator. Further, this means that the older adults who have more positive expectations are more confident in their abilities in taking good care of their health.

The indirect effect of the independent variable towards the dependent variable with the mediator variable was tested using bootstrapping (*N* = 10,000 samples). These results indicate that the indirect effect coefficient is significant, *β* = .09, 95% CI [0.01, 0.20]. This mediation analysis is significant because the confidence interval does not include 0.0. Therefore, we can say that we are 95% confident that the true indirect effect is positive. This is considered to be a medium effect size (Preacher & Kelley, 2011) which means that the effects of expectations regarding aging towards health-promoting lifestyle is mediated by self-efficacy in health. Expectations regarding aging can directly affect health-promoting lifestyle but it can also have an indirect effect through self-efficacy in health.

## Discussion

For the regression analysis, the results suggest that older adults who have positive expectations about aging and who are more confident in taking good care of their health are more likely to engage in health promotion activities.

Moreover, the study suggests that having a positive expectation of aging can have favorable effects on both self-efficacy in health and health-promoting lifestyle. This effect on self-efficacy in health can in turn affect the engagement in health-promoting lifestyle. The results suggest that older adults who have positive expectations about their aging are more likely to engage in health promotion activities when it is coupled with the belief of being more confident in engaging in these activities.

The results are supported by previous research that identified the link with aging expectations and engagement in health promoting activities (Breda & Watts, 2017). Earlier research suggests that people who keep negative aging expectations underestimate their capacity to participate in physical activity, consequently accepting a more inactive lifestyle (O’Brien Cousins, 2000, 2003). Similarly, older persons who believe that old age would result in unavoidable physical decline did not engage in physical activity (O’Brien Cousins, 2000, 2003). Sarkisian et al. (2005) studied the association between aging expectations and physical activity and found a significant positive relationship between positive aging expectations and aerobic activity. People with negative aging expectations were less likely to engage in physical activity. Therefore, negative aging expectations may be a hindrance to physical activity in older adults (Sarkisian et al., 2005).

The present-day influence of these enduring negative aging beliefs on physical activity can be seen by older adults supporting the idea that physical activities are an inappropriate behavior for their age group due to the perceived associated risks (O’Brien Cousins, 2000). Both real and perceived health- and death-related concerns have also been seen to decrease levels of physical activities (O’Brien Cousins, 2000, 2003). Negative aging stereotypes and attitudes could lead to disengagement in physical activities and to the rejection of its usefulness and benefits (Meisner 2012).

On the other hand, people who think that old age is not associated with physical declines and diseases will confirm this identity by engaging in a healthy lifestyle. Results from the German Aging Survey proposed that middle-aged persons with more positive perceptions of age-related growth and decline engaged in sports more frequently, as long as they were healthy enough to be able to do so (Wurm et al., 2010 as cited in Westerhof & Wurm 2015).

Based on the results another factor that influences engagement in health promoting activities would be the belief of successfully doing something or self-efficacy. People with low self-efficacy perceptions are uncertain with their capacity to succeed and do not believe they have the ability for them to attain their goals. People with high self-efficacy perceptions believe that they have high capability; they accept more challenging objectives than those with low self-efficacy perceptions. Since accepting more challenging goals is associated with better performance (Locke & Latham, as cited in Brown, 2015), persons with high self-efficacy perceptions have a tendency to perform well on tasks than those who are uncertain of their capability to succeed.

A study states that older adults who have high self-efficacy and participated in physical activity reported lower levels of depression and increased levels of life satisfaction (Barriopedro et al., as cited in Singh et al., 2010). Furthermore, people will select a behavior grounded on their expectation about their own capability to do a given action and this perceived capability plays a significant role in improving mental well-being & functioning and exercise adherence (Davis et al., as cited in Singh et al., 2010).

The current study was limited by the relatively small sample size and the use of convenience sampling. There may be a possibility that the population may not represent the average, older adult in the Philippines. But to enhance representativeness even with the use of non-probability sampling, there was recruitment in their houses and during the monthly meetings of the senior citizen association.

The findings validate the assumption that aging expectations influence engagement in healthy lifestyles. Older adults who have positive expectations about their aging and believe that they can take care of their health are more likely to engage in health promotion activities.

Based on the findings, it is recommended that older adults and the general public should be educated with the difference between expected and abnormal changes related to aging or old age. There should be greater emphasis on educating people with regards to the negative physical aging stereotypes related to aging. By being able to distinguish between the two, older adults will be able to reduce negative stereotypes about aging, thus being able to promote engagement in a healthy lifestyle even in old age. Society should correct the misconceptions about aging and a positive image of aging should be promoted. There should also be activities that would reinforce a positive belief in being able to engage in health-promoting activities. Furthermore, programs should be designed in a way that will enhance both aging expectations and self-efficacy in order for older adults to engage in more health-promoting activities. Studying older adults’ expectations and its link to health behaviors can provide crucial information on how to design appropriate health-promoting programs. These activities could be done by non-government organizations, local government units, and health care practitioners especially nurse gerontologists.

Lastly, future researchers may explore the possible factors that may influence aging expectations and self-efficacy. Studies could also be done to determine other factors that influence engagement in health-promoting behaviors. A longitudinal study, to establish the clear temporal and causal link between expectations and engagement in health promoting behaviors should also be done. Researchers may conduct studies that would also include older adults with specific health conditions to determine if there are differences with their aging expectations due to their health status. Further, studies may be conducted to various age groups aside from older adults to explore the possible influence of historical and environmental differences towards development of aging expectations. Lastly, future researchers may also conduct studies that would investigate effective interventions to enhance aging expectations and self-efficacy in health.

## Declaration of Conflicting Interests

I confirm that I have no potential conflicts of interest concerning this research.

## Funding

The author acknowledges receipt of financial support for the research, authorship, and/or publication of this article through a dissertation grant from the Commission on Higher Education of the Philippines.
